# DENTALE: a validated Dentofacial EvaluatioN Tool to standardize radiological Assessment of Late dental and maxillofacial adverse Effects following treatment for pediatric rhabdomyosarcoma

**DOI:** 10.3389/fped.2026.1797184

**Published:** 2026-05-22

**Authors:** Koen B. Krommenhoek, Reinier C. Hoogeveen, E. Foster-Thomas, Willem M. M. Fennis, Frédérique J. M. San Giorgi, Matthew Krasin, Mark N. Gaze, Olga Slater, Gillian A. Whitfield, Daniel J. Indelicato, Henry C. Mandeville, Shermaine Pan, Marianne C. Aznar, Johannes H. M. Merks, Alfred G. Becking, Marinka L. F. Hol

**Affiliations:** 1 Department of Oral and Maxillofacial Surgery, Academic Centre Dentistry Amsterdam (ACTA), Amsterdam, Netherlands; 2 Department of Oral and Maxillofacial Surgery, Amsterdam UMC University of Amsterdam, Amsterdam, Netherlands; 3 Princess Máxima Centre for Paediatric Oncology, Utrecht, Netherlands; 4 Department of Oral Radiology and Digital Dentistry, Academic Centre for Dentistry Amsterdam (ACTA), University of Amsterdam & Vrije Universiteit Amsterdam, Amsterdam, Netherlands; 5 NIHR Doctoral Fellow in Restorative Dentistry, Manchester University NHS Foundation Trust, Manchester, United Kingdom; 6 Division of Cancer Sciences, School of Medical Sciences, Faculty of Biology, Medicine and Health, The University of Manchester, Manchester, United Kingdom; 7 Department of Oral and Maxillofacial Surgery, Prosthodontics and Special Dental Care, University Medical Centre Utrecht, Utrecht, Netherlands; 8 Department of Radiation Oncology, St Jude Children's Research Hospital, Memphis, TN, United States; 9 Department of Oncology, University College London Hospitals NHS Foundation Trust, London, United Kingdom; 10 Department of Paediatric Oncology, Great Ormond Street Hospital for Children NHS Foundation Trust, London, United Kingdom; 11 Department of Proton Beam Therapy, Christie Proton Beam Therapy Centre, Manchester, United Kingdom; 12 Radiation Oncology, University of Florida College of Medicine, Jacksonville, FL, United States; 13 Department of Radiotherapy, The Royal Marsden Hospital and the Institute of Cancer Research, Sutton, United Kingdom; 14 Division of Imaging and Oncology, University Medical Centre Utrecht, Utrecht University, Utrecht, Netherlands; 15 Department of Otolaryngology and Head and Neck Cancer, University Medical Center Utrecht, Utrecht University, Utrecht, Netherlands

**Keywords:** dental effects, dental radiograph, head and neck radiation therapy, pediatric oncological patients, rhabdomyosarcoma, survivorship

## Abstract

**Objectives:**

The treatment of childhood cancers causes significant late dental adverse effects, which are evident on radiographic imaging and potentially affect future function. Currently, no standardized tool exists for objectively assessing these effects. This study aimed to develop and validate a new radiographic evaluation tool, “DENTALE,” to guide dental care for survivors and aid research on late dental effects.

**Methods:**

This cross-sectional validation study employed a systematic tool development approach, following established guidelines. The tool was developed through orthopantomogram (OPG) assessment by three dentists using 10 cases of head and neck rhabdomyosarcoma survivors with a wide range of dental abnormalities. Development validation was conducted through the evaluation of 14 OPGs by three independent dentists. Interobserver reliability was assessed using intraclass correlation coefficients (ICCs). Content validity was established through expert consensus, and clinical utility was evaluated using sensitivity and specificity analyses for referral decision-making. This was later validated in a larger cohort (*n *= 83) and subsequently adapted.

**Results:**

The key anatomical features evaluated by the tool are as follows: the morphological condylar process, mandibular ascending ramus, alveolar process, tooth presence, and tooth morphology. Interobserver reliability demonstrated good to excellent agreement (ICC: 0.857–0.939, 95% CI: 0.66–0.98). Using a threshold score of 16 points, the tool achieved 100% sensitivity and 90.0% specificity for specialized dental care referral decisions, with positive and negative predictive values of 94.6% and 100%, respectively.

**Conclusions:**

DENTALE offers a validated and practical radiographic scoring system for OPGs, enabling both clinicians and researchers to assess objectively late dental adverse effects following childhood cancer treatments.

**Clinical significance:**

This tool supports evidence-based referral to specialized dental healthcare, thereby supporting patient management. Furthermore, by standardizing methodology and reporting research on dental late effects, DENTALE can facilitate comparisons across treatments and treatment modalities.

## Introduction

**Figure 1:**
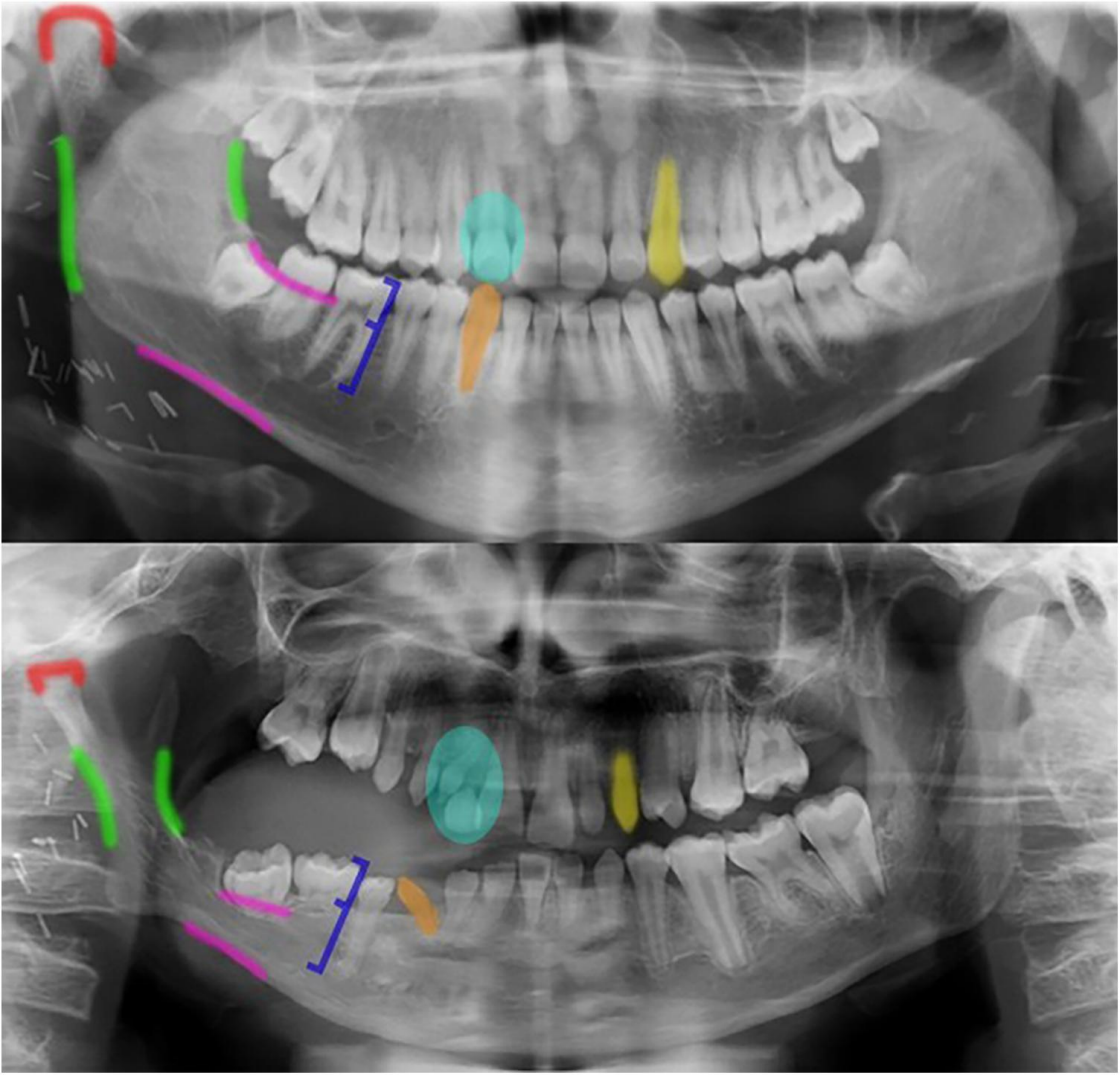
Comparative orthopantomograms (OPGs) of pediatric head and neck rhabdomyosarcoma survivors [14 years old (above) and 13.5 years old (below) at the time of the OPG] demonstrating the spectrum of treatment-related dental and skeletal sequelae. The upper image shows minimal to no adverse effects, while the lower image reveals multiple abnormalities, including impaired root development (orange; normal vs. affected roots), altered crown-root ratios (dark blue line, normal vs. increased crown-root ratio), delayed tooth eruption (light blue circle; age-appropriate vs. delayed eruption), condylar flattening (red line; normal rounded vs. flattened condyle), underdeveloped ascending mandibular ramus (green lines), alveolar process reduction (pink lines, normal vs. reduced aspect of the alveolar bone), and altered crown morphology (yellow; normal vs. crown hypoplasia).

The overall survival of childhood cancer patients is increasing due to multimodal treatments, consisting of chemotherapy (CT), radiation therapy (RT), immunotherapy, and/or surgery. The potential late adverse effects (AEs) for survivors include, among many others, abnormalities in the development of the jaws (maxilla, mandible) and the teeth. With increasing survival, dental and maxillofacial adverse effects are becoming more apparent and have consequently initiated further research interest. Children receiving CT for acute leukemia have been shown to have delayed dental development, microdontia, and hypoplasia (1). Late dental adverse effects due to head and neck RT include agenesis, microdontia, root issues, enamel defects, and delayed eruption (2). These issues are compounded by RT damage to the growing craniofacial skeleton, which remains a significant problem (3). Studies examining the combined late effects of CT and RT report alterations in root development, crown-root malformations, unerupted teeth, enamel hypoplasia, discoloration, agenesis, microdontia, and macrodontia (4). Furthermore, oral late effects such as hyposalivation and susceptibility to mucosal infections have been described in childhood cancer survivors (5, 6). Figure 1, an orthopantomogram (OPG), depicts some previously described late adverse effects.

Standardized definitions for late adverse effects are crucial for both quality of care and for research purposes. Descriptions in the literature of dental AEs are usually based on either the Common Terminology Criteria for Adverse Events (CTCAE) scoring for periodontal disease and tooth development disorders; the Decayed, Missing and Filled Tooth index (DMFT) grading for the effects of caries; or radiological evaluation using the Hölttä Defect Index (DeI) grading for the effects of radiotherapy, which is mainly focused on root development (7–9); these are further specified in the Supplementary Data 1. However, these tools focus on certain aspects of dental health or tooth development, omitting an overall view of the bony structures (jaws), oral health, and dental status.

We aim to develop and validate a tool for survivors of childhood cancer, which we named DENTALE (Dental EvaluatioN Tool for Adverse Late Effects, Supplementary Data 2), to assess the radiographically observable late dental adverse effects of cancer treatments on OPGs. If successful, DENTALE could help determine the severity of late adverse effects and guide the referral to specialized dental care. Such early referral may reduce the risk of complex dental problems in the future. DENTALE would become a standardized tool for assessing late dental adverse effects related to antineoplastic treatments.

## Materials and methods

### Development of the tool

**Figure 2:**
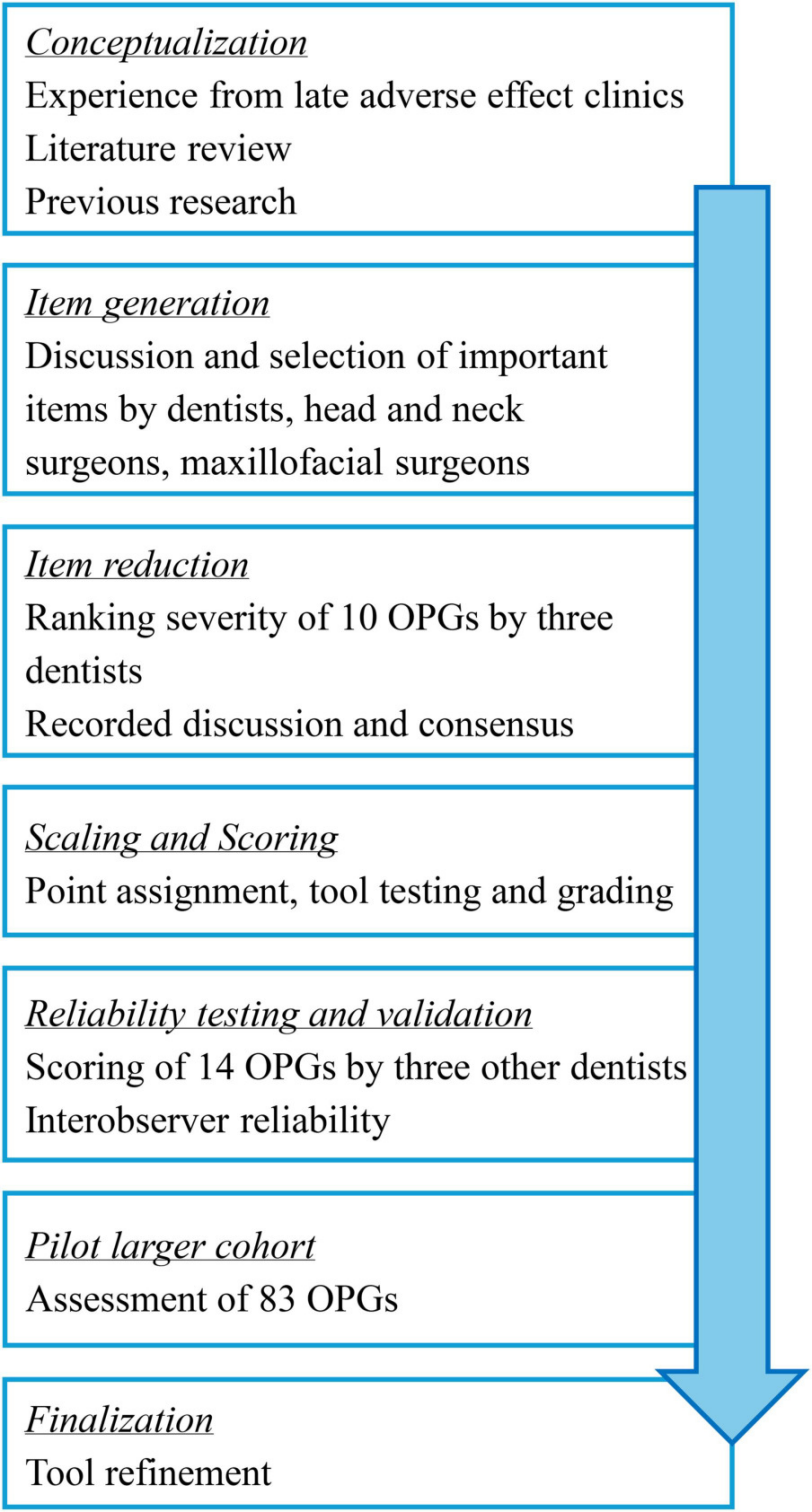
Flowchart of the development of the tool based on the steps described in *Health Measurement Scales: A Practical Guide to Their Development and Use* (5th edition) (10), with the addition of a pilot in a larger cohort.

The development of DENTALE followed the steps outlined in Figure 2 from “Health Measurement Scales: A Practical Guide to Their Development and Use” (5th edition) (10).

### Conceptualization

A cohort of head and neck rhabdomyosarcoma survivors, all treated at the Emma Children's Hospital, University Medical Centre Amsterdam (EKZ-AUMC) between 1 January 1990 and 1 January 2014, was identified and their OPGs were extracted to develop DENTALE. Inclusion criteria required a history of systemic CT and local RT to the head and/or neck. Additional surgery was not considered an exclusion criterion. To be eligible, the OPGs had to be taken at least 2 years post-treatment, and survivors had to have their permanent dentition clinically or radiographically present in order to capture late adverse effects. Out of 103 OPGs, 10 OPGs were selected based on diversity in treatment history, tumor location, and age at diagnosis to ensure adequate representation of the cohort.

### Item generation, reduction, scaling, and scoring items

Two dental faculty members and one orthodontist from the Department of Oral Radiology (EB, MvS, RH) independently ranked the selected 10 OPGs based on the severity of dental sequelae and assessed the need for specialized dental care. Referral to specialized dental care is warranted when the late effects of cancer treatment can no longer be managed within general dental or orthodontic practice based on the nature and severity of the late effects and the complexity of the required treatment. Indications include severely impaired root development, hypodontia or anodontia, ectopic tooth eruption, progressive tooth loss, impaired jaw growth or micrognathia following irradiation, and situations requiring reconstructive treatment, such as implant rehabilitation in irradiated bone or prosthetic reconstruction after jaw defects. Such cases require a multidisciplinary approach beyond routine dental care and hence warrant referral to specialized dental healthcare. Furthermore, scoring items were selected based on reviewed studies, a recorded structured discussion following the individual rankings, and clinical expertise from our late adverse effect clinics (11). A multidisciplinary team of four dentists, one head and neck surgeon with experience in OPG assessments and survivorship assessments, and two maxillofacial surgeons finalized item selection. Patient input was not collected as the tool focuses on the radiograph rather than on patient-reported outcomes.

Ratings were based on the bony and dental structures visible on the OPG, as well as the anticipated dental care needs. Evaluation criteria were grouped into the following two categories: general and tooth-specific aspects. The general aspects were mandible contours, condylar head morphology, mandibular body height, bone density and the alveolar process; tooth-specific items were crown and root morphology, crown-root ratio, age-appropriate eruption, and tooth wear.

Asymmetry, chewing ability, and aesthetics were discussed and excluded due to a lack of consensus on relevance and insufficient reliability of OPG assessment for these factors. Disturbances that require clinical assessment of the teeth were excluded, as the tool assesses radiological findings only.

Each selected item for OPG assessment was assigned a weighted score based on the importance and previously mentioned rank of the OPG based on the expert opinions and the preliminary literature review. The bony aspects were considered first. The condyle plays a crucial role in mandibular function; its underdevelopment may restrict mouth opening, impair speech and mastication, and may contribute to craniofacial asymmetry through altered mandibular growth and alignment (12). Although some degree of mandibular asymmetry is considered physiological, significant asymmetry can negatively impact facial aesthetics and the outcomes of orthodontic treatment (13). It is important to note that only the bony contours of the condyle can be reliably assessed on OPG imaging. Non-osseous structures such as the articular disc, joint capsule, pterygoid muscles, bilaminar zone, and articular cartilage cannot be evaluated using an OPG. In addition, symptoms such as pain, trismus, and facial asymmetry may have extra-articular causes that are not detectable on OPGs. Therefore, the condyle was awarded three points. Although the mandibular ramus angle influences occlusion and facial symmetry (14), its limited functional relevance warranted an assignment of only one point.

The alveolar processes are essential for dental stability, supporting the permanent dentition and assisting interventions such as prosthetic reconstruction and orthodontic treatment (15, 16). Given the significance of the bony aspects of the alveolar processes, one point was assigned to each sextant, resulting in a maximum of six points if the entire dentition is affected.

Tooth position (excluding bucco-lingual) and tooth presence can be identified on OPGs (17, 18). The scoring for missing teeth was measured under the item “Presence” and was weighted based on functional and aesthetic relevance. The canines, which are crucial for reconstruction and long-term orofacial function (19), received eight points each; the incisors were valued at six points each as they are notable aesthetically. Finally, the premolars and molars received four points each. Further tooth-specific aspects were assigned one point per tooth. Tooth wear, if visible on an OPG, despite its multifactorial etiology, was included due to its potential to reflect functional asymmetry associated with osseous or muscular abnormalities. It was therefore scored one point per sextant.

The complete scoring system for DENTALE is presented in Supplementary Data 2*.*

For the overall dental assessments, OPGs are suitable for screening, whereas intraoral radiographs provide greater detail of tooth structure **(**17). DENTALE exclusively focuses on the permanent dentition as it reflects long-term treatment effects and impacts the quality of one’s entire life. Eruption ages were based on Logan and Kronfield and Lunt and Law (20, 21). Abnormal development of the crown, which can be identified on an OPG, is focused on size, specifically microdontia (abnormally small) as well as macrodontia (abnormally large). However, only gross deviations in size that are easily discernible by clinical judgement are often described (18). In the literature, clear definitions of microdontia are rare; therefore, our tool defined microdontia by the crown shape that is expected for the age of the survivor, root structure that is well-defined and distinct, and a cusp count that is normal for the type of tooth. In the case of caries, crown shape restoration as a result of a dental filling is included in the tool. Usually, in adults, a variety of crown-to-root ratios have been described for different teeth, with maxillary canines having a ratio of 1.9, mandibular second molars a ratio of 2.0, maxillary first molars 1.64, and mandibular central incisors 1.59 (22). Crown-to-root ratio has been considered essential for assessing the structural integrity of teeth (23), and a favorable crown-to-root ratio indicates a better foundation for restorative procedures (22). Tooth eruption should correspond to the survivor's age, as delayed eruption may indicate a future need for intervention (20). DENTALE did not account for altered eruption patterns, such as those observed in malnourished patients (24). This finding was excluded as it was unexpected, given that previous studies have only reported delayed tooth eruption (5, 25), and altered eruption sequences have not been observed in our late effects clinic during 10 years of systematic follow-up. Root importance is described in Hölttä's Defect Index (DeI) (9). However, the accuracy of using OPGs for tooth length assessment in orthodontic patients is debatable, as OPG imaging direction can cause the roots and crowns to appear equally longer or shorter than their actual length. Therefore, a crown-root ratio of 1:1 was chosen as the threshold for abnormal development.

The assessment of third molars was excluded due to the high baseline prevalence of complications in the general population; for example, approximately 72% of Swedish adults aged 20–30 have at least one impacted wisdom tooth (26). Dental caries, a multifactorial condition, although associated with chemo- and radiation therapy in both children and adults, was not included as a parameter. The effects of caries on crown morphology have already been accounted for in the tool. Moreover, caries can typically be managed in general dental practice and rarely warrants specialist input. The aesthetic aspects were also excluded, as OPGs do not reliably capture tooth alignment or color, both essential to aesthetic evaluation. In DENTALE, the assessment of asymmetry on OPGs was excluded, as significant condylar contour and ramus asymmetries already indicate a developmental imbalance, while minor variations may be a physiological and functional asymmetry that cannot be reliably assessed with OPGs.

This weighting system results in a scoring range of 0–158, meaning that no adverse effects of treatment were seen in permanent teeth when an OPG is scored 0. A maximum of 158 points indicates that all teeth are missing, both mandibular condyles and ascending rami are affected, and all sextants show reduction of alveolar bone; in this scenario, tooth wear scoring is inapplicable.

### Reliability, validity testing, and statistical analysis

Three dentists from diverse clinical backgrounds (primary dental care, specialized dental care) and geographical locations (the Netherlands, the United Kingdom) (EF-T, FG, WF) independently rated a new set of OPGs using the newly developed tool. This set consisted of 10 different OPGs selected from the 103 OPGs previously mentioned, plus four OPGs that had been used by the first three dentists to verify their initial judgments. The intraclass correlation coefficient (ICC) was used to assess the interobserver reliability of the continuous total score, where 0 indicates no reliability and 1 indicates perfect reliability. To determine the optimal cutoff point for referral, validation was performed by testing various thresholds to compare the consensus among the three dentists on whether (based on the OPG alone) the survivor should be referred to specialized dental care or could continue to be followed up in standard dental care. This was evaluated using different thresholds to identify the most clinically appropriate cutoff point, prioritizing patient safety while minimizing over-referral.

### Pilot testing on a larger cohort

A dentist with expertise in orthodontics and oral and maxillofacial radiology (RH) conducted a pilot study on 83 survivors from our international cohort, all of whom were treated with CT and RT, scoring OPGs and assessing the need for specialized care. Sensitivity and specificity were evaluated, and the tool was refined accordingly.

## Results

### Reliability, validity testing, and statistical analysis

The tool’s interobserver reliability was tested, as shown in Table 1.

**Table 1:** Interobserver reliability per item for assessment at timepoint 1 and assessment at timepoint 2.

Interobserver reliability with the interclass correlation						
Assessed items	ICC timepoint 1	Sig.	95% CI	ICC timepoint 2	Sig.	95% CI
General aspects	0.787	<0.001	0.56–0.92	0.928	<0.001	0.84–0.97
Tooth aspects	0.871	<0.001	0.65–0.96	0.926	<0.001	0.82–0.97
**Total assessment**	**0**.**857**	**<0**.**001**	**0.66–0.95**	**0**.**939**	**<0**.**001**	**0.93–0.98**

Bold values represent total assessment ICCs.

For the general aspects, an interobserver reliability of 0.787 in the first round and 0.928 in the second round was found, which indicates good to excellent reliability. The aspects of teeth, crown, crown-root, eruption, and root varied less in reliability, as calculated using ICCs. For the tooth aspects, the interobserver reliability differed in a range of 0.871–0.926; the total assessment interobserver reliability showed a correlation of 0.857 and 0.939 in the first and second rounds, respectively.

### Pilot testing on a larger cohort

In the validation phase of a larger cohort (*n* = 83 rhabdomyosarcoma survivors), we compared the tool's recommendations with expert clinical judgment for referral to specialized dental healthcare. An expert clinician independently reviewed all cases and determined which survivors required specialized dental care based on a comprehensive radiological assessment.

Several thresholds based on DENTALE (14, 16, 18, and 20 points) were tested. Eventually, a threshold of 16 points in DENTALE was considered optimal, as this threshold correctly identified all the patients whom the specialists deemed to require specialized dental care (100% correct referral rate). With the threshold of 16 points, 50 true positives were identified, indicating appropriate referrals to specialized dental care, and two false positives, representing unwarranted referrals. Three false negatives occurred, while 28 true negatives were correctly classified as not requiring referral. This yielded a sensitivity of 100% and a specificity of 67.2%. The positive predictive value (PPV) was 96.3%, and the negative predictive value (NPV) was 100%, indicating strong diagnostic accuracy in distinguishing cases requiring general vs. specialized dental management based on OPG assessment. Assessors reported a 5-min time requirement to use DENTALE, with assessment time decreasing as familiarity with the scoring form increased.

The tool correctly identified the majority of cases requiring referral. However, three survivors (3.6%) were initially misclassified by the tool as not requiring referral, despite expert opinion that they needed specialized dental care. Analysis of these misclassified cases revealed that they all had borderline scores, with dental abnormalities all confined to a single sextant. To address cases such as these, the tool was refined and now includes the following criterion: if >10 points are scored within one sextant, an additional five points must be added to the total score. This adjustment ensured the detection of localized but radiographically significant pathology.

After re-evaluation, sensitivity remained at 100% and specificity improved to 90.0%, with three false positives. The revised PPV was 94.6%, and the NPV remained 100%. The dentist noted three exceptions. First, one survivor was only 5 years old at the time of the OPG, which is too young to assess late effects in permanent teeth. However, DENTALE could still be used to document their current dental status. Second, two survivors had teeth extracted for orthodontic reasons, which are not considered late effects.

## Discussion

### Main findings and significance

This paper describes the development and validation of DENTALE, a dental assessment tool for identifying late adverse effects in jaws and teeth following antineoplastic treatment for childhood cancer. The agreement between observers was excellent (ICC=0.85–0.93). DENTALE, therefore, is a reliable tool and an important advancement in both clinical decision-making and structured research.

DENTALE supports recommendations from key survivorship organizations, such as PanCare and the Children's Oncology Group (COG), which advise referral to specialized dental care but do not specify when to refer. The PanCare guidelines recommend referral to a specialist dentist or orthodontist for significant dental issues related to prior treatment, such as caries, developmental problems, xerostomia, or periodontal disease (27). According to the COG, children who received over 10 Gy of radiation to the head/brain, neck, spine, or total body should have annual oral examinations and biannual dental check-ups and cleanings (28). A baseline orthopantomogram is advised before dental procedures to assess root development, along with consultation with an orthodontist experienced in treating irradiated childhood cancer survivors. DENTALE standardizes the assessment of dental abnormalities and aids in the evaluation of bone development issues, thereby supporting standardized and comprehensive follow-up care. Since childhood cancer is rare, the majority of dentists may rarely see these survivors, so it is essential to raise awareness of the issues survivors may experience and when specialized dental care is needed.

### Strengths and limitations

The tool was developed through a unique collaboration between dentists, head and neck surgeons, and maxillofacial surgeons. The development process of this tool followed standard procedures for creating health measurement scales, with all items supported by literature. The predefined disturbances, as described in the Materials and Methods section, were not further adjusted after piloting the tool. The tool was re-evaluated for ease of use and potential gaps. Validity testing showed a strong correlation with clinical outcomes, while reliability was confirmed through interobserver assessments. Given its development and validation in a population of childhood cancer survivors, the tool accounts for the effects of chemotherapy, radiotherapy, and surgery, possibly making it applicable for more than rhabdomyosarcoma survivors.

However, there are some limitations. While our validation included experts with different areas of expertise in pediatric dentistry, we did not specifically test the tool with primary care dental practitioners. Our experts work in both primary care and specialized care. The specialists on our panel ensured the tool had good clinical depth and was based on evidence. As the research to develop DENTALE was performed on a cohort of patients who were at least 2 years post-treatment, it consisted mainly of children aged 10 years or older. However, a young child who is not yet dentally mature may develop significant problems and should be seen by a specialized dentist sooner rather than later. Even though the scoring system was developed to grade permanent teeth only, which is important to note, it has also been used in dentally immature children. This, however, requires dentists to grade the permanent teeth and their state of development according to the attached development table. While this is the first comprehensive scoring tool for late dental adverse effects, we lacked information on the dental history of our study population, such as whether teeth had been extracted before cancer treatment or if teeth were missing beforehand (i.e., congenitally missing). This issue also affects the general population, so it may not necessarily reflect a late effect. Although the tool was developed with input from head and neck surgeons and maxillofacial surgeons, whose clinical experience with this patient population informed parameter selection, validation was purposefully performed among dentists. The intended end-users are general and primary care dentists operating outside the hospital setting and dental researchers; its reliability for other clinicians remains unestablished, potentially limiting broader clinical application. Furthermore, the limited sample size precluded final validation of the additional 5-point scoring system for sextants scoring more than 10 points, affecting only three survivors in our cohort and highlighting the need for larger-scale validation studies. In addition, the tool focuses on radiological features and does not capture current oral conditions that are clinically diagnosed, such as enamel defects (enamel hypoplasia and hypomineralization), caries, periodontitis, or dental plaque, as well as chewing and asymmetry in function, which would be obtained during a physical examination. Future research could benefit from a complementary clinical tool to assess overall clinical oral health. Finally, the tool was purposely developed without direct patient input. While our weighting criteria indirectly reflect patient impact, incorporating patient-reported outcomes regarding the relative burden of different anomalies would be valuable for future tool refinement.

### Recommendations

The intended use of this tool is 2-fold: first, clinical implementation to ensure that childhood cancer survivors receive appropriate dental care by facilitating referral to specialized dental healthcare when necessary, and second, for research purposes, by promoting uniformity in the reporting of late adverse dental effects, thereby supporting and enhancing research collaboration. Although this tool was validated in a cohort receiving both RT and CT, future validation in an acute lymphoblastic leukemia cohort with CT-only treatment would strengthen these findings.

### Clinical use

This tool aims to help identify childhood head and neck cancer survivors requiring specialist dental care beyond routine 6-monthly checkups. Using DENTALE requires approximately 5 min, which makes it clinically applicable. With a high sensitivity of 100% and specificity of 90.3%, it ensures early referrals without under-treatment. The OPG is not recommended as a routine screening tool, but rather as a targeted diagnostic instrument for patients with a known history of oncological treatment that may affect dental development. We encourage the incorporation of this tool, DENTALE, into international follow-up guidelines for pediatric cancer survivors by dentists. In addition, we recommend specialist dental evaluations until at least the age of 10 years, as by this age the transition from primary to permanent dentition is largely underway or complete for most teeth, allowing a first meaningful assessment of the permanent dentition on an OPG. Therefore, we encourage pediatric clinicians to create awareness among their patients and their patients’ dentists.

### Research use

The lack of standardized tools for assessing late adverse dental effects limits comparisons across studies and the development of evidence-based treatment guidelines. A standardized tool is essential for consistent scoring and meaningful comparisons, especially with small sample sizes. This approach will aid in dose-effect modeling, help identify critical treatment thresholds, and inform future updates to radiation dose limits for dental tissues.

## Conclusion

DENTALE offers a practical, standardized, and validated scoring system for clinicians and researchers to objectively assess the radiographically observable late dental adverse effects following antineoplastic treatments in childhood cancer survivors. It provides a clear framework for determining when to refer patients to specialized dental care, enhancing patient care and promoting better interdisciplinary communication. Furthermore, it enables multidisciplinary research collaboration.

## Data Availability

The datasets presented in this article are not readily available because data are available upon reasonable request and in consultation with the corresponding author and the local data center of the Princess Máxima Center for Pediatric Oncology. Requests to access the datasets should be directed to m.l.f.hol-9@prinsesmaximacentrum.nl.
