# Supplementary material for: DENTALE: a validated Dentofacial EvaluatioN Tool to standardize radiological Assessment of Late dental and maxillofacial adverse Effects following treatment for pediatric rhabdomyosarcoma
Source: Front Pediatr. 2026 May 22;14:1797184. doi: 10.3389/fped.2026.1797184 (PMC13236946; doi:10.3389/fped.2026.1797184)
Supplement: Supplementary file 1 [file Supplementaryfile1.pdf]

Supplementary data 1

	**Common Terminology Criteria for Advserse Effects (CTCAE v5.0)** (1)	**Höltta Defect Index (DeI)** (2)	**Decayed Missing Filled Teeth (DMFT)** (3)	**DENTALE**
**Assessment**	Clinical	Radiograph	Clinical / Radiograph	Radiograph
**Tooth aspects**				
Caries	X	-	X	-
Eruption disorder	-	-	-	X
Missing tooth	-	-	Missing tooth	Missing incisor, canine, or (pre)molar
Periodontal disease	X	-	-	Alveolar bone reduction
Toothache	X	-	-	-
Tooth development disorder	X	Crown-root disturbance, microdontia, aplasia	Aplasia	Crown, crown-root, root
Tooth discoloration	X	-	-	-
Tooth filling	-	-	Filling	Crown shape correcting restoration
Tooth infection	X	-	-	-
Tooth wear	-	-	-	X
**Bony aspects**				
Mandibular condyle	-	-	-	X
Mandibular ascending ramus	-	-	-	X
Osteonecrosis of the jaw	X	-	-	-

**CTCAE v5.0 – Tooth and jaw related CTCAE terms** (1)					
CTCAE term	Grade 1	Grade 2	Grade 3	Grade 4	Grade 5
**Dental caries**	One or more dental caries, not involving the root	Dental caries involving the root	Dental caries resulting in pulpitis or periapical abscess or resulting in tooth loss		
Definition: A disorder characterized by the decay of a tooth, in which it becomes softened, discolored and/or porous.					
**Periodontal disease**	Gingival recession or gingivitis; limited bleeding on probing; mild local bone loss	Moderate gingival recession or gingivitis; multiple sites of bleeding on probing; moderate bone loss	Spontaneous bleeding; severe bone loss with or without tooth loss; osteonecrosis of maxilla or mandible		
Definition: A disorder in the gingival tissue around the teeth					

**Tooth development disorder**	Asymptomatic; hypoplasia of tooth or enamel	Impairment correctable with oral surgery	Maldevelopment with impairment not surgically correctable; limiting self care ADL		
Definition: A disorder characterized by a pathological process of the teeth occurring during tooth development.					
**Tooth discoloration**	Surface stains				
Definition: A disorder characterized by a change in tooth hue or tint.					
**Toothache**	Mild pain	Moderate pain; limiting instrumental ADL	Severe pain; limiting self care ADL		
Definition: A disorder characterized by a sensation of marked discomfort in the tooth.					
**Tooth infection**		Localized; local intervention indicated (e.g., topical antibiotic, antifungal, or antiviral)	IV antibiotic, antifungal, or antiviral intervention indicated; invasive intervention indicated	Life-threatening consequences; urgent intervention indicated	Death
Definition: A disorder characterized by an infectious process involving a tooth.					
**Osteonecrosis of jaw**	Asymptomatic; clinical or diagnostic observations only; intervention not indicated	Symptomatic; medical intervention indicated (e.g., topical agents); limiting instrumental ADL	Severe symptoms; limiting self care ADL; elective operative intervention indicated	Life-threatening consequences; urgent intervention indicated	Death
Definition: A disorder characterized by a necrotic process occurring in the bone of the mandible.					

Höllta's Defect Index (DeI) (2002) (2)

The DeI was developed to describe the severity of damage to the dentition. It combines the abnormalities in root-to-crown ratio, microdontia and agenesis, and subsequently representing total damage to the permanent dentition as a single index figure. The higher the index, the stronger the deviations from normal tooth development (normal DeI = 0).

Each tooth was first categorized using the following criteria:

ND not determined
- (a) developing teeth with an unclear final outcome
- (b) missing teeth not categorized in the aplasia group because of young age
- (c) teeth not reliably seen on radiograph

D0 R/C ratio >1.6; no disturbance
D1 R/C ratio 1.2–1.6; mild disturbance
D2 R/C ratio 0.9–1.1; severe disturbance
D3 R/C ratio <0.9; very severe disturbance or arrested root development
D4 microdontia; exceptionally small tooth
D5 aplasia; missing tooth

In the aplasia category D5, a tooth was not considered missing before the following ages:

first premolar:	< 5 years
second premolar:	< 6 years
second molar:	< 6 years

third molar: < 13 years

The DeI is finally calculated as follows: (nD1 × 1) + (nD2 × 2) + (nD3 × 3) + (nD4 × 4) + (nD5 × 5), where n is the number of teeth in the respective disturbance category D1, D2, D3, D4 or D5

Decayed, Missing, and Filled Teeth – index (1930) (3)

The Decayed, Missing, and Filled Teeth (DMFT) index, established in the 1930s, serves as the primary global measure for assessing caries experience at the population level. This metric calculates the total number of an individual's decayed, missing, and filled permanent teeth, yielding scores from zero to 28 (excluding third molars).

Formula: DMFT = decayed teeth + missing teeth + filled teeth (maximum = 28)

Supplementary data 2 - DENTALE

**Dentofacial evaluation tool for assessment of late adverse effects “DENTALE”**	
This tool is developed to identify radiological dentofacial abnormalities after cancer treatment for childhood cancer survivors. Please read the supplementary explainer for the definitions used for this tool.	
Patient ID: ______<br>Sex: F / M / X	Age at evaluation: ______<br>Date of evaluation: - -
**Pretreatment dental history**	**Treatment characteristics**
Caries treatment:	Age at diagnosis:
Extracted teeth:	Tumour type:
Orthodontic treatment:	Tumour localization:

**Chemotherapy** □ yes □ no<br>*Protocol*:	**Radiation** □ yes □ no<br>*Protocol*:	**Surgery** □ yes □ no<br>*Resected tissues*:

**General aspects**									**score**
Mandibular condyle	**0**<br>both sides normal		**3**<br>one side affected:		□ □<br>R L			**6**<br>both sides affected	
Mandibular ascending ramus	**0**<br>both sides normal		**1**<br>one side affected:		□ □<br>R L			**2**<br>both sides affected	
Bony aspect of the alveolar process	**0**	**1**	**2**	**3**		**4**	**5**	**6**	
	score 1 per affected sextant, if affected, which sextant (cross in presence)								
Tooth wear	**0**	**1**	**2**	**3**		**4**	**5**	**6**	
	score 1 per affected sextant, if affected, which sextant (cross in presence)								

Presence

sext 1 - alveolar □, tooth wear □ | sext 2 - alveolar □, tooth wear □ | sext 3 - alveolar □, tooth wear □

18	17	16	15	14	13	12	11	21	22	23	24	25	26	27	28
□ E<br>□ C<br>□ CR<br>□ R	□ E<br>□ C<br>□ CR<br>□ R	□ E<br>□ C<br>□ CR<br>□ R	□ E<br>□ C<br>□ CR<br>□ R	□ E<br>□ C<br>□ CR<br>□ R	□ E<br>□ C<br>□ CR<br>□ R	□ E<br>□ C<br>□ CR<br>□ R	□ E<br>□ C<br>□ CR<br>□ R	□ E<br>□ C<br>□ CR<br>□ R	□ E<br>□ C<br>□ CR<br>□ R	□ E<br>□ C<br>□ CR<br>□ R	□ E<br>□ C<br>□ CR<br>□ R	□ E<br>□ C<br>□ CR<br>□ R	□ E<br>□ C<br>□ CR<br>□ R	□ E<br>□ C<br>□ CR<br>□ R	□ E<br>□ C<br>□ CR<br>□ R
□ E<br>□ C<br>□ CR<br>□ R	□ E<br>□ C<br>□ CR<br>□ R	□ E<br>□ C<br>□ CR<br>□ R	□ E<br>□ C<br>□ CR<br>□ R	□ E<br>□ C<br>□ CR<br>□ R	□ E<br>□ C<br>□ CR<br>□ R	□ E<br>□ C<br>□ CR<br>□ R	□ E<br>□ C<br>□ CR<br>□ R	□ E<br>□ C<br>□ CR<br>□ R	□ E<br>□ C<br>□ CR<br>□ R	□ E<br>□ C<br>□ CR<br>□ R	□ E<br>□ C<br>□ CR<br>□ R	□ E<br>□ C<br>□ CR<br>□ R	□ E<br>□ C<br>□ CR<br>□ R	□ E<br>□ C<br>□ CR<br>□ R	□ E<br>□ C<br>□ CR<br>□ R
48	47	46	45	44	43	42	41	31	32	33	34	35	36	37	38

sext 6 - alveolar □, tooth wear □ | sext 5 - alveolar □, tooth wear □ | sext 4 - alveolar □, tooth wear □

		**number**	**score**
Missing incisors	+ 6 points per incisor		
Missing canines	+ 8 points per canine		
Missing molars	+ 4 points per (pre)molar		

**Tooth specific aspects**	**0**	**1**	**score** (0-28)
Eruption (E)	Normal	Abnormal eruption direction of permanent tooth, or for age unfitting non eruption of the tooth.	
Crown (C)	Normal	Abnormal morphology, crown shape correcting restoration, microdontia (crown), macrodontia (crown), different crown shape than expected.	
Crown-root ratio (CR)	Normal	Severely underdeveloped roots, meaning the crown > root (ratio > 1.0).	
Root (R)	Normal	V-shaped root, short root, hypoplastic root. Inappropriate for the age and the tooth root has not been well-formed.	
One sextant *most* affected and ≥ 10 points (including presence)? Yes = + 5 points, no = 0 points			

**Total score:**<br>*referral to specialized dentist needed if ≥ 16 points*	

Supplementary explainer "DENTALE"	
Definitions	
Mandibular condyle	*Consider normal if:* The mandibular condyle is rounded to oval-shaped, with symmetrical condyles on both the right and left sides, and no bony changes such as flattening or erosion.<br>*Consider affected if:* The mandibular condyle is not rounded or oval-shaped, if the condyles on the right and left sides are asymmetrical, or if there are bony changes such as flattening or erosion.
Mandibular ascending ramus	*Consider normal if:* The ramus height and breadth are as expected based on the participant's age and sex.<br>*Consider affected if:* There is asymmetry, underdevelopment, or visible evidence of surgical or orthodontic interventions to restore function.
Alveolar process	*Consider normal if*: The distance between the alveolar process and the cemento-enamel junction is 1.0 mm or less.<br>*Consider abnormal if*: The distance is more than 1.0 mm.
Tooth wear	*Consider normal if*: There are no signs of flattening of occlusal surfaces.<br>*Consider affected if:* reduced height or flattening of occlusal surfaces that indicate wear.
Presence	*Assess the presence of a permanent tooth*: Consider it present if the tooth can be identified, whether it has erupted or not, and even if it is still covered by the deciduous dentition. Consider it not present if the tooth cannot be identified.
Crown	*Consider normal if:* The crown shape is as expected for the age, the root structure is well-defined and distinct, and the cusp count is normal.<br>*Consider affected if:* The crown shape is abnormal, there are changes due to fillings, or there is microdontia or macrodontia of the crown, or if the crown shape differs from what is expected, or a crown-shape correcting restoration.
Crown-root ratio	*Consider normal if:* The root is larger than the crown, taking into account that the root should be fully developed and appropriate for the age.<br>*Consider abnormal if:* The tooth is severely underdeveloped, meaning the crown is as large as or larger than the root.
Eruption	*Consider normal if:* The tooth has erupted in an acceptable direction, appropriate for the age.<br>*Consider abnormal if:* The permanent tooth has an abnormal eruption direction or if the tooth has not erupted in a manner fitting for the age.
Root	*Please note it is likely the root is abnormal in case the crown-root ratio was abnormal already. Please score abnormal again for root than.*<br>*Consider normal if:* The tooth development is appropriate for the age and the tooth root is well-formed.<br>*Consider abnormal if:* The root is V-shaped, short, or hypoplastic.

Chronology of human permanent dentition development*					
**Tooth**	**Tooth No**	**Calcification begins at**	**Crown (enamel) completed at**	**Root completed at**	**Eruption**
**Maxillary teeth**					
Central incisor	11, 21	3 - 4 mo	4 - 5 y	10 y	7 - 8 y
Lateral incisor	12, 22	10 - 12 mo	4 - 5 y	11 y	8 - 9 y
Canine	13, 23	4 - 5 mo	6 - 7 y	13 – 15 y	11 - 12 y
First premolar	14, 24	1 ½ - 1 ¾ y	5 - 6 y	12 - 13 y	10 - 11 y
Second premolar	15, 25	2 – 2 ¼ y	6 - 7 y	12 - 14 y	10 - 12 y
First molar	16, 26	birth	2 ½ - 3 y	9 – 10 y	6 – 7 y
Second molar	17, 27	2 ½ - 3 y	7 - 8 y	14 -16 y	12 – 13 y
**Mandibular teeth**					
Central incisor	31, 41	3 – 4 mo	4 – 5 y	9 y	6 - 7 y
Lateral incisor	32, 42	3 – 4 mo	4 – 5 y	10 y	7 – 8 y
Canine	33, 43	4 -5 mo	6 – 7 y	12 – 14 y	9 – 10 y
First premolar	34, 44	1 ¾ - 2 y	5 - 6 y	12 – 13 y	10 – 12 y
Second premolar	35, 45	2 ¼ - 2 ½ y	6 – 7 y	13 - 14 y	11 – 12 y
First molar	36, 46	birth	2 ½ - 3 y	9 – 10 y	6 – 7 y
Second molar	37, 47	2 ½ - 3 y	7 -8 y	14 – 15 y	11 – 13 y

*Logan, W.H., & Kronfeld, R. (1933). Development of the human jaws and surrounding structures from birth to age fifteen. *Journal of the American Dental Association*, *20*, 379-428,
Schour, I., & McCall, J.O. (1944). Chronology of the human dentition. In B. Orban (Ed.), *Oral histology and embryology* (p. 240). C.V. Mosby

References

1. Cancer Institute N. Common Terminology Criteria for Adverse Events (CTCAE) Common Terminology Criteria for Adverse Events (CTCAE) v5.0 [Internet]. 2017. Available from: https://www.meddra.org/

2. Hölttä P, Alaluusua S, Saarinen-Pihkala UM, Wolf J, Nyström M, Hovi L. Post-transplant complications Long-term adverse effects on dentition in children with poor-risk neuroblastoma treated with high-dose chemotherapy and autologous stem cell transplantation with or without total body irradiation. Bone Marrow Transplant [Internet]. 2002;29:121–7. Available from: www.nature.com/bmt

3. Shulman JD, Cappelli DP. Epidemiology of Dental Caries. In: Prevention in Clinical Oral Health Care. 2008. p. 2–13.
